# Saponins from *Panax japonicus* attenuate cognitive impairment in ageing rats through regulating microglial polarisation and autophagy

**DOI:** 10.1080/13880209.2021.1961824

**Published:** 2021-08-17

**Authors:** Xue-Jiao Pi, Qing-Qing Zhao, Jin-Xin Wang, Xu-Lan Zhang, Ding Yuan, Shan-Shan Hu, Yu-Min He, Chang-Cheng Zhang, Zhi-Yong Zhou, Ting Wang

**Affiliations:** a Academy of Nutrition and Health, Hubei Province Key Laboratory of Occupational Hazard Identification and Control, Wuhan University of Science and Technology, Wuhan, China; b Department of Pharmacy, College of Medicine, Wuhan University of Science and Technology, Wuhan, China; c College of Medical Science, Three Gorges University, Yichang, China

**Keywords:** Neuroinflamamtion, cognitive function, microglia, M1-like phenotype, M2-like phenotype

## Abstract

**Context:**

*Panax japonicus* is the dried rhizome of *Panax japonicus* C.A. Mey. (Araliaceae). Saponins from *Panax japonicus* (SPJ) exhibit anti-inflammatory and antioxidative effects.

**Objective:**

To explore the neuroprotective effect of SPJ on natural ageing of rat.

**Materials and methods:**

Sprague-Dawley (SD) rats 18-month-old were divided into ageing control, ageing treated with SPJ 10 or 30 mg/kg (*n* = 8). Five-month-old rats were taken as the adult control (*n* = 8). Rats were fed regular feed or feed containing SPJ for 4 months. Cognitive level was evaluated by Morris water maze (MWM) test. The mechanisms of SPJ’s neuroprotection were evaluated by transmission electron microscope, western blot analysis, and immunofluorescence *in vivo* and *in vitro*.

**Results:**

SPJ attenuated ageing-induced cognitive impairment as indicated by elevated number of times crossing the target platform (from 1.63 to 3.5) and longer time spent in the target platform quadrant (from 1.33 to 1.98). Meanwhile, SPJ improved the morphology of microglia and synapse, and activated M2 microglia polarisation including increased hippocampus levels of CD206 (from 0.98 to 1.47) and YM-1 (from 0.67 to 1.1), and enhanced autophagy-related proteins LC3B (from 0.48 to 0.82), Beclin1 (from 0.32 to 0.51), Atg5 (from 0.22 to 0.89) whereas decreased p62 level (from 0.71 to 0.45) of ageing rats. *In vitro* study also showed that SPJ regulated the microglial polarisation and autophagy.

**Discussion and conclusions:**

SPJ improved cognitive deficits of ageing rats through attenuating microglial inflammation and enhancing microglial autophagy, which could be used to treat neurodegenerative disorders.

## Introduction

Neurodegenerative disorders (NDDs) are often the consequence of degenerative response to numerous systemic-level processes, but the exact cause has not been fully elucidated, however, ageing is widely considered to be the greatest risk factor (Zia et al. 2021). It is predicted that the number of elderly people in the world will increase by 21% in the next 50 years (Tricco et al. 2012). The World Health Organisation (WHO) predicts that by 2040, ageing-related neurodegenerative disease such as Alzheimer’s disease (AD), Parkinson’s disease (PD), and amyotrophic lateral sclerosis (ALS) will overtake cancer as the second leading cause of death after cardiovascular disease (Gammon 2014). The link between ageing and increased inflammation has been well established. Both men and women over 65 years old have elevated serum levels of IL-6, TNF-α, and IL-18 (Ferrucci et al. 2005; Nilsson et al. 2020). Substantial evidence suggests that systemic low-grade inflammation contributed to the development of brain degeneration, especially with ageing progresses (Goldberg and Dixit 2015; Więckowska-Gacek et al. 2021). In the brain, the theory of ‘hypothalamic microinflammation’ mainly emphasises the fundamental role of inflammation in ageing-related NDDs development (Zhang et al. 2013, 2017). In general, inflammation represents a key regulator in both ageing and degenerative disorders (Lutshumba et al. 2021). Therefore, manipulating age-related inflammatory mechanisms may delay the development of NDDs (Guo et al. 2015).

As mononuclear phagocytes residing in the central nervous system (CNS) parenchyma, microglia are a crucial part of the CNS immune system and play a primary role in tissue injury, repair and regeneration (Smith et al. 2012; Hu et al. 2015; Fan and Pang 2017; Wang et al. 2019). Once activated by external stimuli, such as pathogens or injuries, resting microglia become polarised. The two extremes of this polarisation are called M1-like and M2-like phenotypes (Hu et al. 2015; Saxena et al. 2021). M1 microglia are usually activated by pro-inflammatory cytokines such as interferon-γ (IFNγ) or endotoxins such as lipopolysaccharide (LPS). Once in M1 polarisation state, microglia produce pro-inflammatory cytokines, nitric oxide (NO), and reactive oxygen species (ROS), resulting in neuron damage and neurogenic disorders (Block et al. 2007; Prinz and Priller 2014). In contrast, M2 microglia are activated by IL-4 and IL-10, which induce neurogenesis and brain tissue regeneration by secreting anti-inflammatory neurotrophic factors such as IL-10, transforming growth factor-β (TGF-β), insulin-like growth factor 1 (IGF-1) and brain-derived neurotrophic factor (BDNF) (Belarbi and Rosi 2013; Prinz and Priller 2014; Colonna and Butovsky 2017). The transition from M2 microglia to M1 microglia is related to the increased injury progression in NDDs (Hu et al. 2015). Therefore, regulating microglial polarisation from M1 towards M2 is considered to be a potential therapeutic strategy for NDDs.

Meanwhile, autophagy is a basic cellular homeostatic mechanism that is essential for controlling inflammatory responses and neurotoxicity in the CNS (Bussi et al. 2017; He et al. 2018). In the autophagic process, damaged organelles and denatured proteins are transported to lysosomes, in which their functional blocks are degraded and recycled via anabolic reactions (Carneiro and Travassos 2013; Kim et al. 2015). Autophagy-related proteins can either induce or inhibit immune and inflammatory responses, as well as immune and inflammatory signals can also regulate autophagy (Saitoh and Akira 2010; Su et al. 2016). Emerging studies demonstrated that autophagy is associated with the phenotypic transformation of microglia (Xia et al. 2016). For instance, both primary and BV2 microglia with autophagy gene knockout showed excessive M1 pro-inflammatory state (Ye et al. 2017; Houtman et al. 2019). However, when treated with various autophagy inducers, the inflammatory response of microglia was significantly inhibited and the M2 phenotypic polarisation of microglia increased (Munz 2016; Ye et al. 2017; Cheng et al. 2020).

*Panax japonicus,* the dried rhizome of *Panax japonicus* C.A. Mey. (Araliaceae), is a common traditional herbal medicine and widely distributed in Japan and southwest China and some ethnic minorities, it is used as a substitute for Ginseng root (Deng et al. 2017). Saponins from *Panax japonicus* (SPJ) are the bioactive rhizome component of *Panax japonicus* (Yuan et al. 2018). Mounting pharmacological studies have shown that SPJ have anti-inflammatory and antioxidative effects, as well as reduce blood lipids and regulate immunity (Wu et al. 2016), among which our previous studies have shown that the anti-inflammatory activity of SPJ is particularly prominent. For example, SPJ reduced the inflammatory response of LPS-induced BV2 microglia and RAW264.7 macrophages (Dai et al. 2014; Tu et al. 2018). We also showed that SPJ improved the cognitive deficits in natural ageing rats and D-galactose induction induced rats (Wang et al. 2015; Deng et al. 2017; Ruan et al. 2019; Wan et al. 2020). However, whether the effect of SPJ on cognitive impairment is related to regulation of microglial inflammatory phenotype and the level of autophagy remains unclear.

## Materials and methods

### Animal treatment

The specific pathogen free male Sprague-Dawley rats (*n* = 32) were purchased from the Experimental Animal Centre of China Three Gorges University in Yichang, Hubei province. Rats were housed in pathogen-free facilities, in a 12 h light/dark cycle in ventilated cages, with access to chow and water *ad libitum*. This study was approved by the China Three Gorges University Council on Animal Care Committee. The handling, experimental procedures, and care of the animals were carried out in accordance with the National Institutes of Health Guide for the Care and Use of Laboratory Animals. Animals were divided into four groups and treated with various regimens: (a) adult control group (*n* = 8, normal feed, 1-month-old rats were purchased and raised to 5-month old); (b) ageing control group (*n* = 8, normal feed, 18-month-old rats were purchased and raised to 22-month old); (c) SPJ 10 mg/kg treated group (*n* = 8, contains SPJ feed, 18-month old rats were purchased and food administration for consecutive 4 months until they were 22-month-old); (d) SPJ 30 mg/kg treated group (*n* = 8, contains SPJ feed, 18-month old rats were purchased and food administration for consecutive 4 months until they were 22-month-old). Briefly, we measured the daily food intake of each rat, and calculated the SPJ content per kilogram of feed based on the average weight and food intake. Rat feed containing SPJ is processed by Beijing Huafukang Biotechnology Co., Ltd. At a predetermined time, rats were anaesthetized with intraperitoneal injecting urethanes. The cortex and hippocampus were snap frozen for further experiments.

### Cell culture and viability assay

BV2 murine microglia cells were obtained from the Cell Resource Centre of the Institute of Basic Medicine, Chinese Academy of Medical Sciences. Cells were maintained in complete DMEM media (10% foetal bovine serum, 100 units/mL penicillin-streptomycin) and cultivated at 37 °C with 5% CO_2_.

The model of LPS activation was established according to the following steps: cells were pre-protected with 1, 5, 25, 50 μg/mL SPJ for 12 h and followed with 1 μg/mL LPS for 12 h. Cell viability was evaluated by MTT. Briefly, cells were seeded in 96-well plates (1 × 10^4^ cells/well) and then pre-incubated with different concentrations of SPJ. After incubation, MTT solution was added and incubated for 4 h, then MTT solution was removed and 200 μL DMSO was added for 15-min incubation. Finally, the absorbance was measured at 570 nm with a microplate reader. Survival ratio of control group was defined as 100% and that of other groups expressed as percentage of control group.

### Drug sources

*Panax japonicus* was harvested from the planting base of Chunmuying in Xuanen County, Enshi City, Hubei Province in October 2016. It was identified by Dr. He Yumin in Key Laboratory of Natural Products Research and Utilisation of Three Gorges University. SPJ were extracted according to the method of our research team (He et al. 2017). Briefly, the root of dried *Panax japonicus* (1000 g) was cut into small pieces and refluxed with 3-fold volume 70% ethanol for 3 times, 2 h each time. The extract was filtered and concentrated to 5 L, purified by M-5 macroporous resin, eluted with double distilled water, and eluted with 70% ethanol when the Molish reaction was negative. The collected eluent was then decompressed to concentrate, followed by freeze-drying. The dried sample was added with water to make a 20 mg/mL solution, and the pH value was adjusted to 10. The extraction rate is 18.4% and the purity is 83.5%.

### Morris water maze (MWM) test

The MWM test was evaluated one week before the rats were killed. Briefly, tests were performed in a tank (120 cm diameter, 50 cm deep) filled with opacified water kept at 22 ± 1 °C. The tank was equipped with a 10 cm diameter platform submerged 1 cm under the water surface. Training includes daily sessions about 2 h for 5 consecutive days. The start positions varied pseudo randomly among the 4 cardinal points. Each trial ended when the animal reached the platform. The mice were gently guided to the platform if they failed to reach the platform in 1 min. After the last training, retention trial was evaluated during probe trial in which the platform was removed (day 6). Animals’ video track and test parameters including latency, swim speed, travelled distance, time spent in quadrant, platform crossings) were automatically calculated.

### Transmission electron microscope

The tissue was cut into 1 mm^3^, fixed with stationary liquid for 24 h at 4 °C and then fixed in 1% osmic acid for another 2 h. After uranyl acetate staining under room temperature and dark condition for 3 h, samples were washed with redistilled water and dehydrated by ethanol. The dehydrated tissue was placed in a mixture of ethanol and epoxypropane (1:2) for 10 min, epoxypropane twice for 10 min, mixture of epoxypropane and embedding medium (1:1) for 40 min, mixture of epoxypropane and embedding medium (1:4) for 3 h, and then embedded with embedding medium at 4 °C overnight and subsequently polymerised at 60 °C for 48 h. After performing histological section, tissue slide was stained with uranyl acetate and lead citrate, and microglia were observed and photographed under transmission electron microscope.

### Western blotting

All tissues or cells were lysed in RIPA buffer, and total protein concentrations were determined with BCA Protein Assay Kit. Total protein was loaded into precast 8%-12% SDS-PAGE gels and then transferred onto PVDF membranes. After that, the membranes were incubated with the following primary antibodies at 4 °C overnight: anti-β-actin (Servicebio, GB11001, 1:1000), anti-YM-1 (Abcam, ab192029, 1:2000), anti-CD206 (Abcam, ab64693, 1:1000), anti-LC3A/B (Cell signalling, #12741, 1:1000), anti-Beclin1 (Abcam, ab207612, 1:1000), anti-ATG5 (Proteintech, 10181-2-AP, 1:1000), anti-p62 (Abcam, ab56416, 1:2000), anti-IL-1β (Abcam, ab9722, 1:1000). The primary antibodies were recycled and membranes were washed 3 times in Tris-buffered saline with Tween-20 (TBST) for 6 min each time. Goat anti-Rabbit IgG and Goat anti-Mouse IgG secondary antibody (Jackson ImmunoResearch, 103069, 1:10,000) were incubated for 1 h at room temperature. After the removal of the secondary antibody, the membranes were washed 3 times with TBST for 6 min each time. Finally, the relative proteins levels were detected by enhanced chemiluminescence reagent and ImageJ software.

### Immunofluorescence

Cells were seeded on the circular cover glass in 24-well plates and were pre-incubated with 25 and 50 μg/mL SPJ for 12 h and followed with 1 μg/mL LPS for another 12 h. After ending the incubation, cells were washed twice with PBS, followed by fixation with 4% paraform and then perforation with 0.1% Triton X-100 for 20 min. After blocking with 1% BSA, cells were incubated with the following primary antibodies at 4 °C overnight: anti-IL-1β (Abcam, ab9722, 1:2000), anti-TNF-α (Santa Cruz, SC-5746, 1:1000), anti-ARG1 (Proteintech, 16001-1-AP, 1:1000), TGF-β (Abcam, ab92486, 1:1000). Following incubation with primary antibody, cells were incubated with FITC-labeled secondary antibodies (Jackson ImmunoResearch) for 1.5 h. After washing, cells were mounted with 40, 60-diamidino-2-phenylindole (DAPI) and Prolong Antifade Reagent (G1401, Servicebio, China). Staining patterns were examined using a scanning fluorescence microscope (Leica Microsystems).

### Statistical analysis

The obtained data are presented as mean ± SEM of at least three independent experiments. Groups were compared using one-way ANOVA with *post hoc* Tukey’s multiple comparisons test. All of the data were analysed with GraphPad Prism 6.0 software. Statistical significance was set at *p* < 0.05 for all the analyses.

## Results

### SPJ reduces cognitive impairment in ageing rats

**Figure 1.**
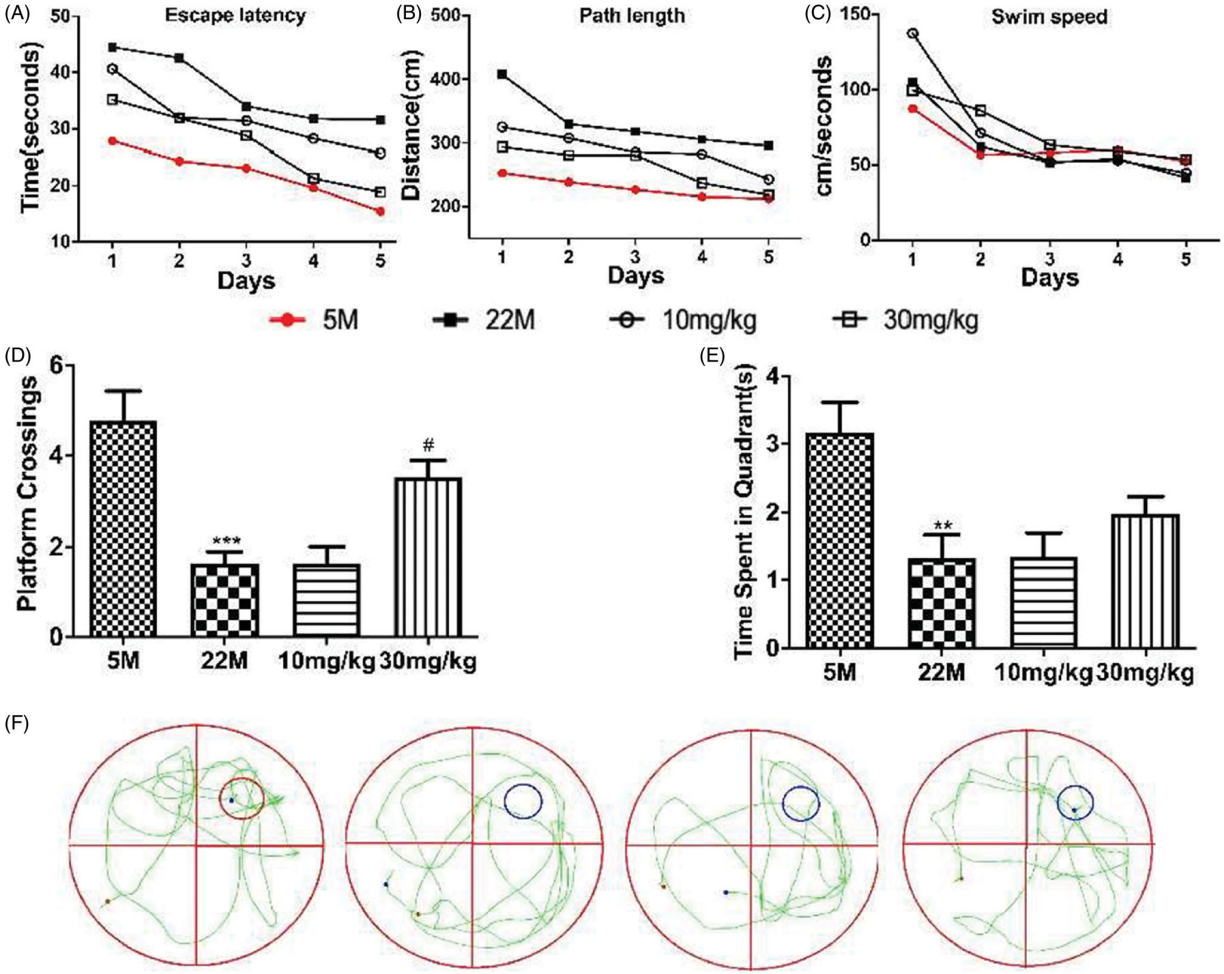
Effect of SPJ on cognitive impairment in ageing rats. (A) Escape latency (B) path length and (C) swim speed of SD rats during learning session. (D) Comparison of numbers of crossing over platform site on day 6. (E) Comparison of time spent in target quadrant on day 6. (F) Representative route on day 6. **p < 0.01 *vs.* 5 M group; ^#^*p* < 0.05 *vs.* 22 M group.

To evaluate the protective action of SPJ on cognitive impairment, rats were tested in the MWM (Figure 1). All rats learned platform position across time during learning session, as demonstrated by decreased latency (Figure 1(A)) or path length (Figure 1(B)) to reach the platform over the 5 days of training. Although impaired learning ability was observed in the ageing rats as compared to the young, SPJ treatment with 10 and 30 mg/kg reduced impaired learning ability relative to the ageing group. It should be noted that the swim speed was comparable in all groups regardless of the treatment with SPJ, indicating no neuromotor differences among the groups (Figure 1(C)). After the last training trial, SPJ-treated rats showed a clear preference for the target quadrant by contrast to the ageing group (SPJ-treated *vs.* ageing rats: platform crossing, *p* < 0.05 (Figure 1(D)), time spent in target quadrant, *p* > 0.05 (Figure 1(E)). These results showed that SPJ alleviated cognitive impairments in memory retention of ageing rats.

### SPJ improves the morphology of microglia and synapses in ageing rats

**Figure 2.**
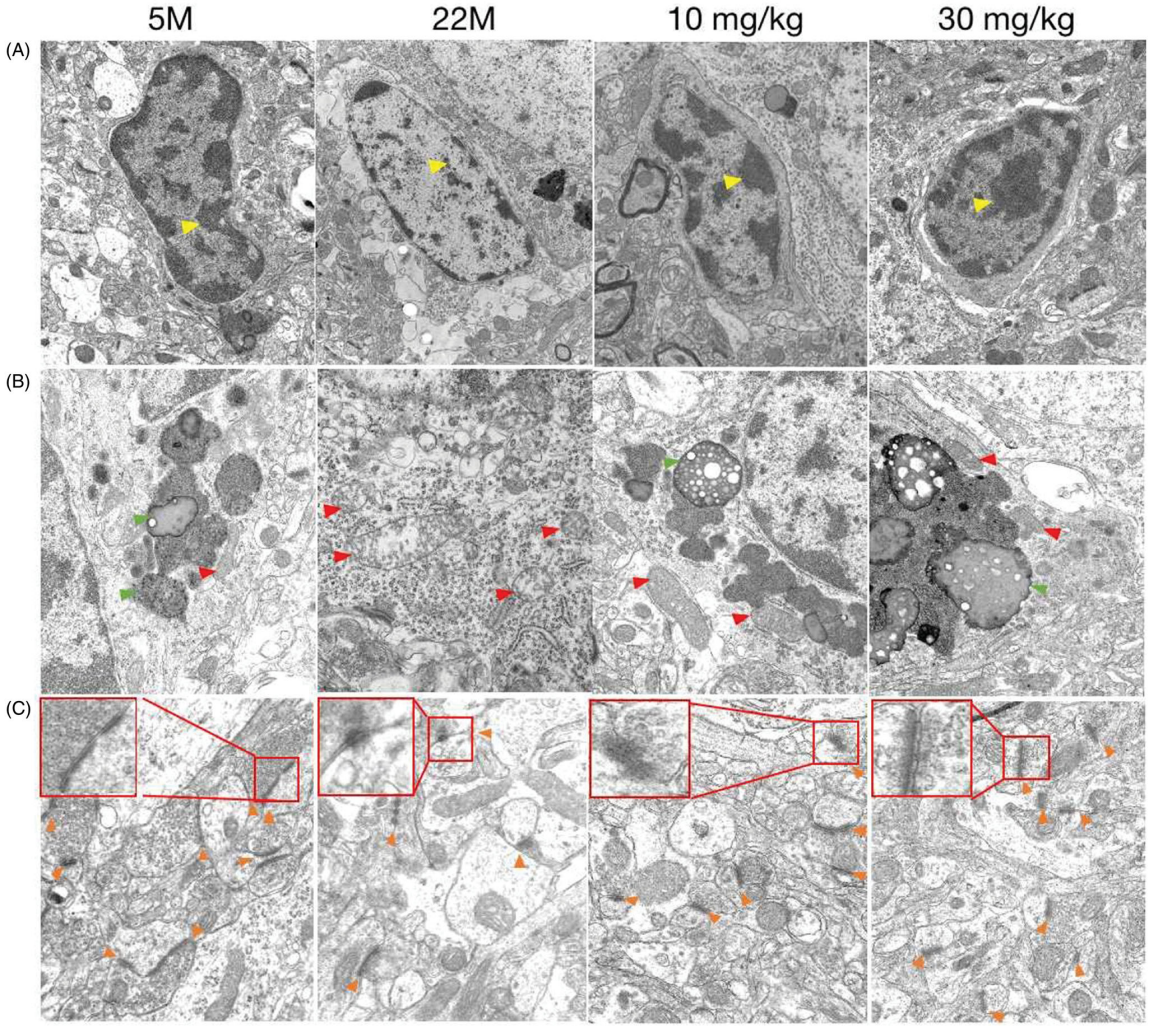
Effect of SPJ on the morphology of microglia and synapses in ageing rats through transmission electron microscope. The morphology of microglial nucleus (A), cytoplasm (B) and neuronal synapses (C) demonstrated that treatment with SPJ at 10 and 30 mg/kg exerted neuroprotective effect by improving damaged microglia and synapses in cortex. Yellow arrows indicate chromatin; Red arrows indicate mitochondria; Orange arrows indicate synapses.

Compared to the 5 M group, cortical microglia in 22 M showed a variable degree of ultrastructural alterations as follows: vacuolisation, chromatin decreased, the nuclear membrane was unclear and the cell organelle (lysosomes, mitochondria) in the cytoplasm were blurred or even disappeared under the transmission electron microscope (Figure 2(A)). Beyond that, mitochondria with variable size (elongated, fragmental size or swelling) and with reduction or distorted/disrupted cristae were found in the ageing microglia (Figure 2(B)). After treatment with SPJ, the pathological phenomena of microglia in the ageing group were significantly improved. The electron density of microglia was high and the organelles were rich in the cytoplasm, as well as the mitochondria were well-structured with clear crista and inner boundary membrane. On the other hand, contrary to 5 M group, the aged group exhibits fewer synapses, blurred structure of the membrane, and significantly fewer synaptic vesicles, all of which have been reversed with intervention of SPJ (Figure 2(C)).

### SPJ promotes M2 polarisation in ageing rats

**Figure 3.**
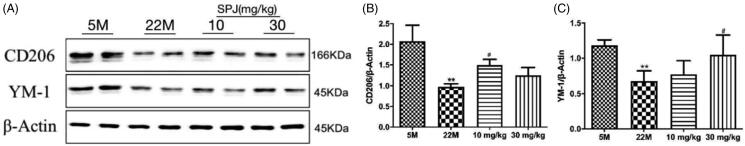
Effect of SPJ on CD206 and YM-1 protein levels in the hippocampus of ageing rats. (A) Representative immunoblot bands of CD206 and YM-1 in hippocampus. (B) and (C) Quantification of CD206/β-actin ratio and YM-1/β-actin ratio. **p < 0.01 *vs.* 5 M group; ^#^*p* < 0.05 *vs.* 22 M group.

To explore the effect of SPJ on microglia polarisation in hippocampus of ageing rats, western blot analysis was used to determine the levels of CD206 and YM-1. The data demonstrated that the protein levels of CD206 and YM-1 were significantly reduced in the ageing rats when compared to the young. However, the above protein expression changes were obviously reversed by SPJ intervention (*p* < 0.05, Figure 3).

### SPJ reverses autophagy impairment in ageing rats

**Figure 4.**
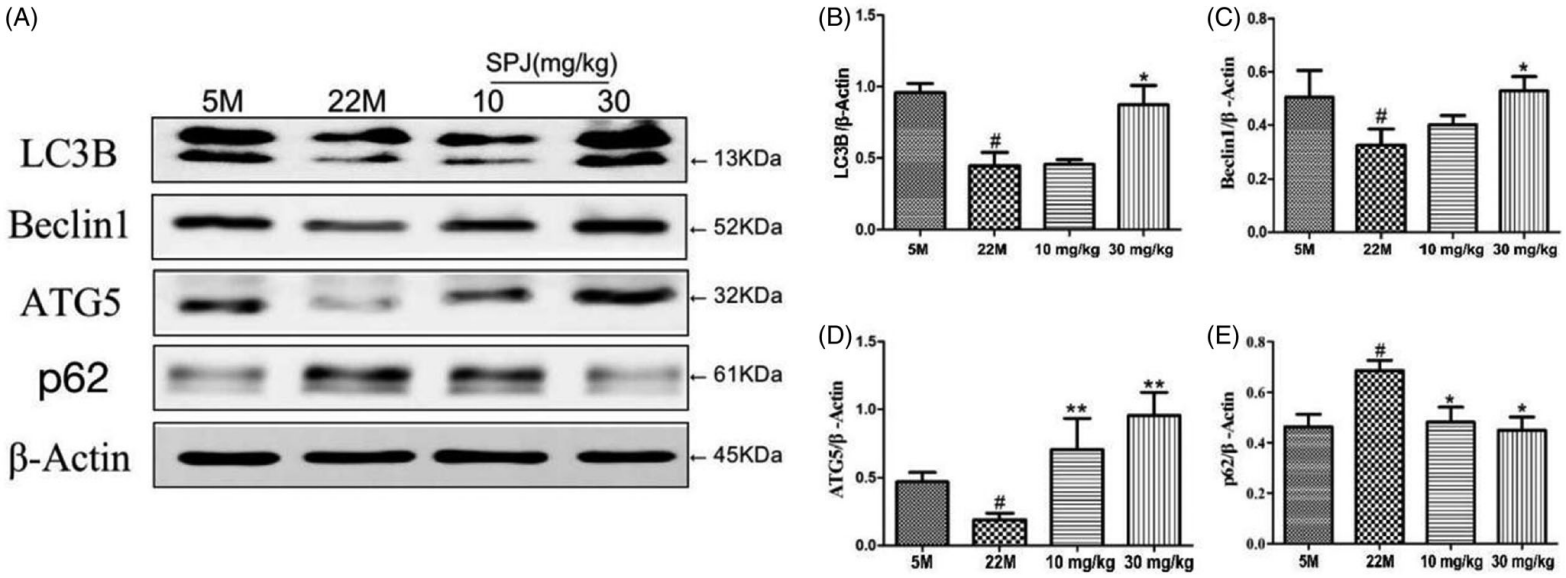
Effect of SPJ on autophagy-related protein levels in the hippocampus of ageing rats. (A) Representative immunoblot bands of LC3, Beclin1, ATG5 and p62 levels in hippocampus. (B–E) Quantification of LC3, Beclin1, ATG5 and p62 after normalisation to β-actin. ^#^*p* < 0.05 *vs.* 5 M group; *p < 0.05 or **p < 0.01 *vs.* 22 M group.

We further examined whether SPJ regulates autophagy progress in ageing rats. Western blotting showed that the levels of LC3B, Beclin1 and ATG5 in the hippocampus of ageing rats were significantly decreased in comparison with the young group (*p* < 0.05, Figure 4(B–D)), whereas the level of p62 was obviously increased (*p* < 0.05, Figure 4(E)). After 30 mg/kg SPJ intervention, the above changes of protein levels were reversed (*p* < 0.05 or *p* < 0.01, Figure 4).

### SPJ reverses LPS-induced M2-to-M1 polarisation in BV2 cells

**Figure 5.**
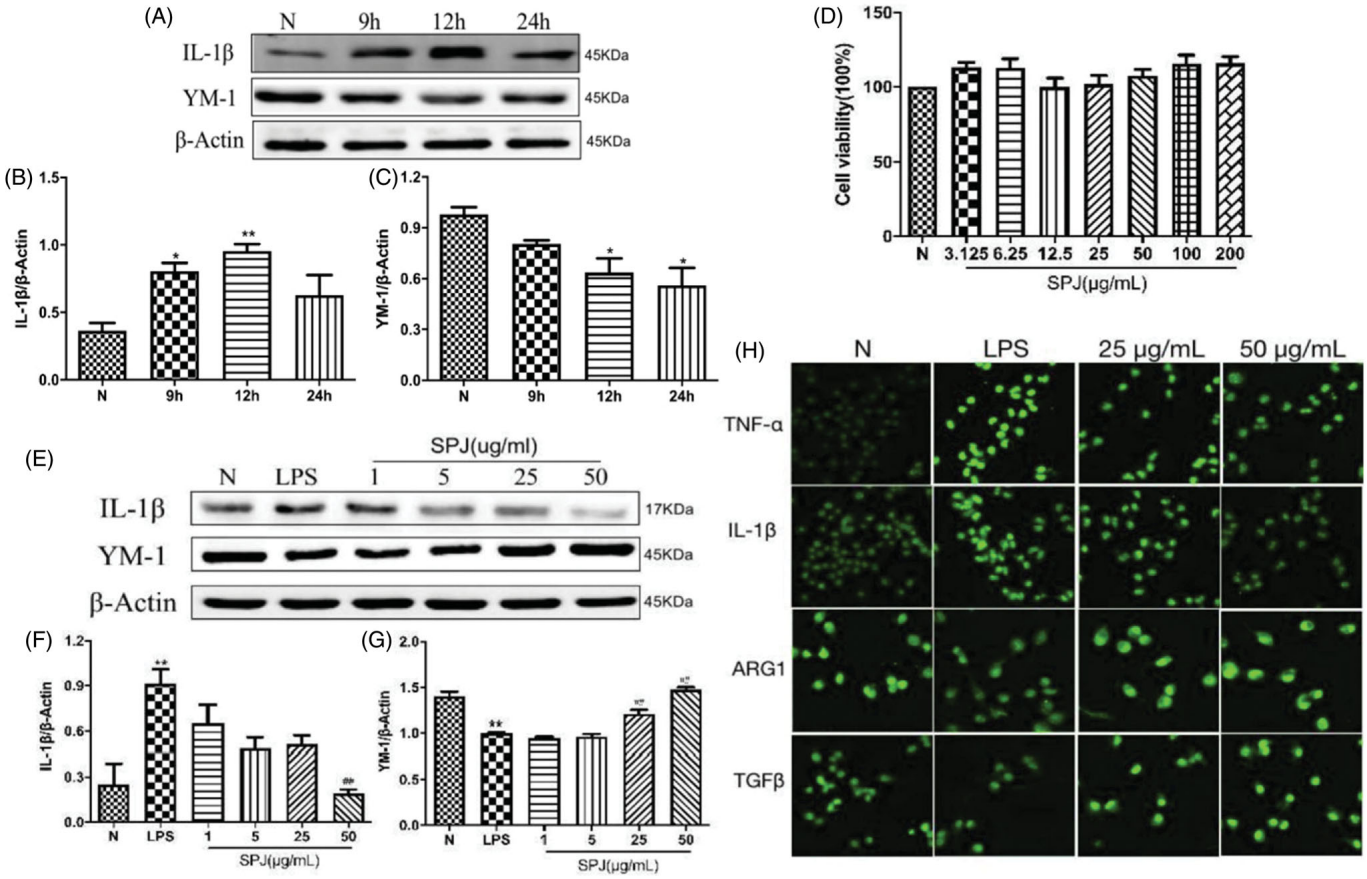
Effect of SPJ on microglial polarisation in LPS-induced BV2 cells. (A) Representative immunoblot bands of IL-1β and YM-1 in cells after stimulation by LPS. (B–C) Quantification of IL-1β and YM-1 after normalisation to β-actin (D) MTT showed that treatment with SPJ alone below the concentration of 200 μM did not induce toxicity in BV2 cells. (E) Representative immunoblot bands of IL-1β and YM-1 in cells upon a SPJ protection for 12 h followed by LPS stimulation. (F–G) Quantification of YM-1and IL-1β after normalisation to β-actin. (H) Confocal images show that the protein levels of TNF-α, IL-1β, ARG1 and TGFβ in BV2 after administrating SPJ in LPS groups. *p < 0.05, **p < 0.01 *vs.* control group; ^##^*p* < 0.01 *vs.* LPS group.

Upon stimulation by LPS for 9, 12 and 24 h, microglia were tested via western blotting to probe protein expressions related to inflammation. Our results found that the level of YM-1 (M2 marker) decreased gradually (Figure 5(A,B), *p* < 0.05), whereas the level of M1 marker IL-1β peaked at 12 h (Figure 5(A–C), *p* < 0.01). The above results showed that LPS enhanced microglial M2-to-M1 polarisation, so 1 μg/mL LPS was selected to stimulate BV2 for 12 h as the model time in the following experiment. Figure 5(D) showed that treatment with SPJ alone below the concentration of 200 μM did not induce toxicity in BV2 cells. Moreover, SPJ inhibited the protein levels of IL-1β in comparison to the LPS groups. Furthermore, the level of YM-1 increased after SPJ incubation (Figure 5(E)). Immunofluorescence further demonstrated an increase in the accumulation of M2 markers, including ARG1 and TGFβ, while SPJ suppressed LPS-induced increase of M1 markers, including TNF-α and IL-1β (Figure 5(H)). These results demonstrate that SPJ promotes microglial M1-to-M2 polarisation.

### SPJ enhanced autophagy inhibited by LPS stimulation in BV2 cells

**Figure 6.**
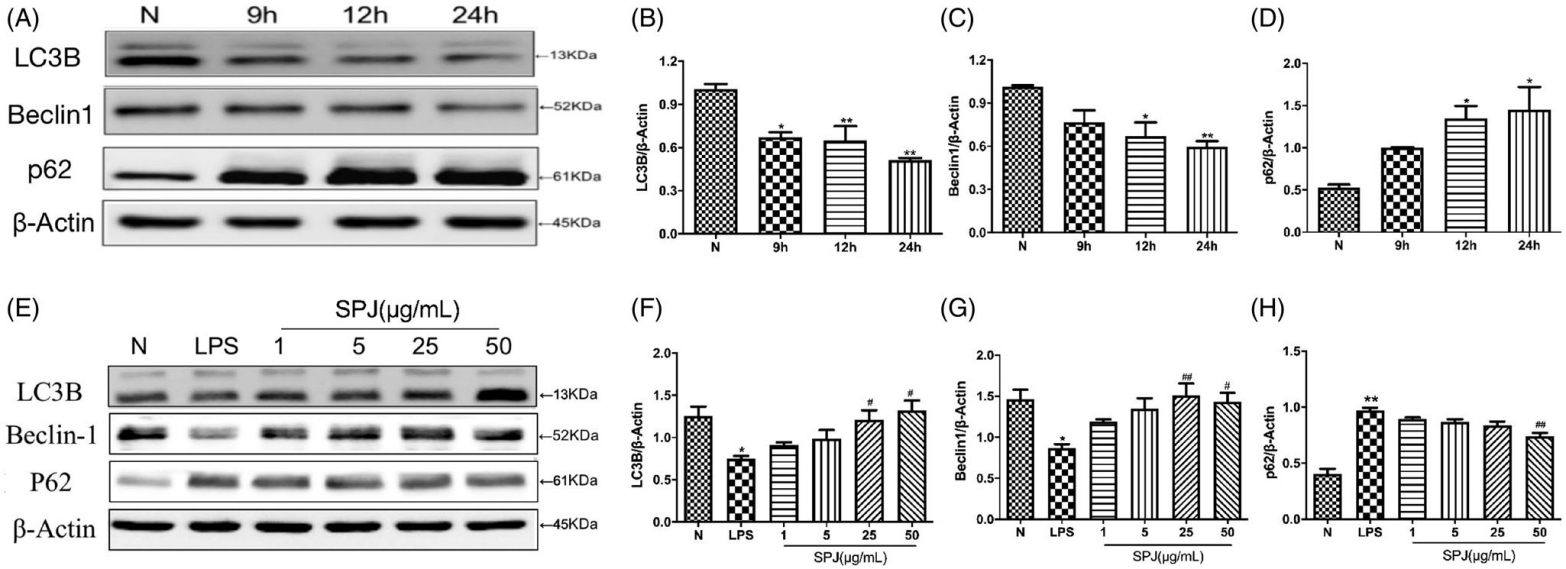
Effect of SPJ on microglial autophagy in LPS-induced BV2 cells. (A) Representative immunoblot bands of LC3B, Beclin1 and p62 in cells after stimulation by LPS. (E) Representative immunoblot bands of LC3B, Beclin1 and p62 in cells upon a SPJ protection for 12 h followed by LPS stimulation. (B–D, F–H) Quantification of LC3B, Beclin1 and p62 after normalisation to β-actin. *p < 0.05, **p < 0.01 *vs.* control group; ^#^*p* < 0.05, ^##^*p* < 0.01 *vs.* LPS group.

Emerging studies have shown the pivotal roles of autophagy involving in microglial inflammation and phenotype shift. In order to evaluate whether activation of inflammation would affect autophagy in BV2 cells, we also detected autophagy related proteins. Since LC3B is a classical marker of autophagy, we evaluated LC3B expression through western blotting and found that it was decreased in cells cotreated with LPS (*p* < 0.01, Figure 6(A–C)). Similar to the LC3B, the level of Beclin1, an important participant in the formation of autophagosomes, significantly decreased after co-incubation with LPS (*p* < 0.01, Figure 6(A–B)). Moreover, LPS treatment significantly increases p62, a protein substrate for autophagy used for monitoring autophagic turnover, in microglial cells (*p* < 0.01, Figure 6(A–D)). The above results reflected impaired autophagy in BV2 with LPS stimulation. However, after treatment the cells with SPJ and LPS, the protein levels of LC3B and Beclin1 increased gradually, whereas the level of p62 gradually decreased (*p* < 0.05 or *p* < 0.01, Figure 6(E–H)). The above findings indicate that SPJ enhance microglial autophagy.

## Discussion

The effects of SPJ on cognitive disorder are gradually illustrated by the behavioural experiment in the model of various CNS disorders (Wang et al. 2015; Deng et al. 2017; Ruan et al. 2019). But the underlying mechanism of SPJ mediated functional change in microglial phenotype remains unclear. This study reveals the consequences of SPJ in ageing-induced cognitive damage. Morris water maze showed that SPJ recovered the cognitive decline compared to the aged group. Transmission electron microscope showed the microglial and synapses’ morphological improvement by lowering the extent for (1) breakage membrane of microglia cell, (2) decreasing mass of chromatin in the nucleolus, (3) injury of organelles in the cytoplasm, and (4) decreasing number and damaging structure of synapses. These results suggested that SPJ may improve cognitive impairment through regulating microglia in ageing mice.

The balance between microglial inflammatory states affects the progression of a multitude of CNS disorders, however, the specific classification of M1 and M2 polarised microglia remains a topic for debate (Hu et al. 2015; Miron et al. 2013; Tian et al. 2016). M1/M2 polarisation of microglia is largely classified by the expression of M1 markers such as IL-1β, TNF-α, COX-2 and iNOS, and M2 markers such as ARG1, YM-1, TGF-β and CD206 (Hu et al. 2015). The polarised M1 or M2 subpopulation can reverse its phenotype and function in order to response to distinct microenvironmental cues. Thus, strategies focus on promoting M1-to-M2 phenotypic conversion of microglia may be regarded as a potential treatment of neuroinflammation-induced damage (Miron et al. 2013; Tian et al. 2016). Actually, emerging evidences indicated that SPJ can reduce the expression of pro-inflammatory factors and NOD-Like receptors-like receptor 3 (NLRP3) inflammatory bodies in hippocampus and cortex of natural ageing rats (Wang et al. 2015; Deng et al. 2017; Ruan et al. 2019). This study further showed that the SPJ induced microglial M2 polarisation in vivo, which indicated by the increase of M2 markers including YM-1 and CD206 in hippocampus. Western blotting and immunofluorescence analyses confirmed that SPJ could promote M1-to-M2 polarisation and exert anti-inflammatory effect in LPS-induced BV2 microglia *in vitro*.

Besides inflammatory response, autophagy is also affected by LPS as reported in a growing number of studies. For example, He et al. (2018) demonstrated that LPS led to p38 MAPK-dependent phosphorylation of ULK1 in microglia, which inhibited ULK1 kinase activity and prevented ULK1 binding to the downstream effector ATG13, finally repressed autophagy in microglia. Lee et al. (2019) found that LPS obviously inhibited autophagy via FOXO3 pathway and impaired the phagocytic capability of microglia. Furthermore, Ye et al. (2020) found that LPS-induced neuroinflammation in microglia resulted from inhibition of autophagic flux through activation of the PI3KI/AKT/MTOR pathway, whereas elevated microglial autophagy alleviated LPS-induced neuroinflammation. Therefore, we evaluated the effect of LPS on autophagy-related proteins in BV2 microglial cell line. As is reported, the expression of LC3B and Beclin1 reduced and the level of p62 elevated in BV2 microglia after incubated with 1 μg/mL LPS for 12 h. The above phenomena can be reversed after the intervention of SPJ, indicating that SPJ can reverse LPS-induced autophagy inhibition in BV2 cells.

Recently, there is increasing evidence that autophagy regulates innate immunity by promoting the transformation of microglia from M1 to M2 (Saitoh and Akira 2010; Sumpter and Levine 2010). In various neuroinflammatory models (e.g., AD, PD, cerebral ischaemia and ALS), the inflammatory bodies in BV2 microglia are activated. Meanwhile, the expression of M1 pro-inflammatory factors is increased, but the M2 anti-inflammatory factors and autophagy are both suppressed (Jin et al. 2018). When autophagy was up-regulated by inhibiting serum or administrating various autophagy activators, microglia exhibited a decreased activation of inflammatory bodies and an increased polarisation to M2 (Cadwell 2016). These improvements were blocked with administration of the autophagy inhibitor 3-MA, which may be related to the regulation of mTOR or TLR2 signal pathway, inhibition of NLRP3 inflammatory bodies or other factors (Li et al. 2016; Xia et al. 2016; de Mattos Barbosa et al. 2018; Han et al. 2019). However, in other autophagy injury models of microglia (e.g., 3-MA treatment, siRNA-Beclin1 or siRNA-ATG5 transfection), NLRP3 signal, MAPKs and nuclear transcription factor (NF-κB) are all activated, and M1 pro-inflammatory cytokines are highly expressed. Such changes are however reversed, accompanied with an increased polarisation of M2, upon treating with various autophagy inducers (e.g., rapamycin, chloroquine and GSK-3β inhibitors) (Zhou et al. 2011; Cheng et al. 2020). Therefore, the inhibition of autophagy may be involved in the over-activation of microglia, while the promotion of autophagy can reduce the microglial pro-inflammatory response. Consistent to this analysis, our results showed that 50 μg/mL SPJ enhanced the autophagy of microglia, while the level of M1 pro-inflammatory factor IL-1β was significantly decreased and the expression of M2 anti-inflammatory factor YM-1 was significantly increased. These suggest that SPJ may exert its neuroprotective effect on microglia by enhancing autophagy that induces the transformation of inflammatory phenotype from M1 to M2.

## Conclusions

Our research proved that SPJ reduced cognitive decline in ageing rats through upregulation of microglial M1 to M2 polarisation resulting in decreasing microglial inflammation and enhancing microglial autophagy ability.
